# Low-Modulus PMMA Has the Potential to Reduce Stresses on Endplates after Cement Discoplasty

**DOI:** 10.3390/jfb13010018

**Published:** 2022-02-04

**Authors:** Susanne Lewin, Peter Försth, Cecilia Persson

**Affiliations:** 1 Department of Materials Science and Engineering, Division of Biomedical Engineering, Uppsala University, 751 21 Uppsala, Sweden; susanne.lewin@angstrom.uu.se; 2 Department of Surgical Sciences, Division of Orthopedics, Uppsala University, 751 85 Uppsala, Sweden; peter.forsth@surgsci.uu.se

**Keywords:** bone cement, discoplasty, PMMA, low-modulus, lumbar spine

## Abstract

Cement discoplasty has been developed to treat patients with advanced intervertebral disc degeneration. In discoplasty, poly(methylmethacrylate) (PMMA) bone cement is injected into the disc, leading to reduced pain and certain spinal alignment correction. Standard PMMA-cements have much higher elastic modulus than the surrounding vertebral bone, which may lead to a propensity for adjacent fractures. A PMMA-cement with lower modulus might be biomechanically beneficial. In this study, PMMA-cements with lower modulus were obtained using previously established methods. A commercial PMMA-cement (V-steady^®^, G21 srl) was used as control, and as base cement. The low-modulus PMMA-cements were modified by 12 vol% (LA12), 16 vol% (LA16) and 20 vol% (LA20) linoleic acid (LA). After storage in 37 °C PBS from 24 h up to 8 weeks, specimens were tested in compression to obtain the material properties. A lower E-modulus was obtained with increasing amount of LA. However, with storage time, the E-modulus increased. Standard and low-modulus PMMA discoplasty were compared in a previously developed and validated computational lumbar spine model. All discoplasty models showed the same trend, namely a substantial reduction in range of motion (ROM), compared to the healthy model. The V-steady model had the largest ROM-reduction (77%), and the LA20 model had the smallest (45%). The average stress at the endplate was higher for all discoplasty models than for the healthy model, but the stresses were reduced for cements with higher amounts of LA. The study indicates that low-modulus PMMA is promising for discoplasty from a mechanical viewpoint. However, validation experiments are needed, and the clinical setting needs to be further considered.

## 1. Introduction

A large part of the population will experience lower back pain (LBP) during their lifetime—under an age of 50 years the prevalence is 70–80%, and over an age of 50 years it increases to over 90% [1]. Often LBP is associated with intravertebral disc (IVD) degeneration [1,2]. A healthy IVD consists of the centrally located hydrostatically pressurized nucleus pulposus, surrounded on the outer periphery by the annulus fibrous. Cartilage and vertebral endplates at the superior and inferior surfaces connect the discs to the vertebrae. With age, the tissues may degrade, the nucleus becomes dehydrated and loses hydrostatic pressurization. In the advanced stage, the substantial degradation of the nucleus may lead to a vacuum space in the disc. This may entail a reduced disc height, increased instability and higher spinal nerve pressure, with resulting leg pain and LBP [2].

Surgical interventions may be needed for degenerated discs with persisting pain. These include interbody fusion using an intervertebral spacer and/or transpedicular stabilization. The procedures require invasive and extensive surgery. For many elderly patients, there is a significant risk of complications due to physical conditions and comorbidities [3]. A less invasive procedure, percutaneous cement discoplasty (PCD), was introduced by Varga et al. [4]. A similar procedure, reported on by Yamada et al., is referred to as percutaneous intervertebral-vacuum polymethylmethacrylate injection (PIPI) [5]. In discoplasty, polymethylmethacrylate (PMMA) is injected into the degenerated disc. Clinical studies have shown that discoplasty significantly reduces lower back and leg pain, but also reduces spinal malalignment [4,6,7,8,9].

Yamada et al. successfully treated LBP by discoplasty in degenerative lumbar scoliosis (DLS) patients. The study contains a comparison between a discoplasty group (*n* = 101) and a group that received non-operative treatment (*n* = 61). The visual analog scale (VAS) for back-pain and the Oswestry disability index (ODI) for the discoplasty group were significantly improved compared to the group with non-surgical treatment. After two years, the proportion of patients reaching a clinically relevant improvement according to MCID (Minimal Clinically Important Difference) was for back-pain 72% after discoplasty compared to 26% in the non-surgical group (*p* < 0.001). The corresponding fractions for ODI were 58% and 17%, respectively (*p* < 0.001). Out of 101 patients, LBP recurred in 35% [9]. Nevertheless, the recurrence came together with the reappearance of bone marrow edema (BME) at the endplates, either at the level of discoplasty or at the level adjacent to discoplasty. BME of the endplate and adjacent areas have been closely associated with LBP [10]. One suggested cause for BME in DLS patients is increased mechanical stresses that induce inflammatory changes in the endplate and its corresponding vertebral bone marrow [10,11]. The endplates have relatively low resistance to mechanical stress and represent the primary site of vertebral damage accumulation [12,13]. Subsidence and implant migration into the endplate can occur when using spinal interbody fusions with cages [14]. In a study with 40 patients, 30% of the cages had migrated into the vertebral endplates at around 2 years follow-up [14]. The experience of fusion cages and endplate subsidence suggest that there is a theoretical risk of endplate fractures and subsidence also after discoplasty.

So far, biomechanical evaluations of discoplasty are rare. One recent experimental study assessed the biomechanical concept of discoplasty [15]. The study tested porcine vertebral segments in flexion, extension, and lateral bending. Three conditions were investigated; an intact disc, after nucleotomy and after discoplasty. The discoplasty recovered the intervertebral posterior height and opened up the neuroforamen, which could be one explanation for the reduction of pain in clinical situations due to reduced pressure on spinal nerves. However, discoplasty (as compared to after nucleotomy) had no significant impact on the spinal mobility (range of motion) and stiffness. The nucleotomy induced bulging of the disc under loading, discoplasty reduced the overall bulging of the disc and very small strains were visible in most of the disc surface [15]. The study indicated that positive clinical results, in terms of increased disc height and increasing the opening of the neuroforamen, could be reproduced experimentally. However, no significant differences were seen between healthy and treated samples for biomechanical parameters such as stiffness or range of motion. Several factors during the experiments could have affected the outcome, such as how the injury in the disc was induced, the amount of cement, the choice of a porcine animal model, etc. To fully understand how PMMA discoplasty affects the spinal biomechanics, more studies in experimental discoplasty models are required. Since experimental models of discoplasty are still under development, computational models would be an interesting approach for theoretical studies of different concepts of discoplasty biomechanics. Interesting topics to study would for example be different types of PMMA cements.

The current standard PMMA cements, which have so far been used in discoplasty, have a much higher elastic modulus (~3000 MPa) than that of the surrounding vertebral bone (10–900 MPa) [16,17,18,19]. More compliant PMMA materials have been developed for use in vertebroplasty, i.e., vertebral bone fracture stabilization. These so called low-modulus PMMA cements have been developed for osteoporotic patients in particular [20]. One such material is a linoleic acid modified PMMA (LA-PMMA), where the additive of linoleic acid essentially works as a plasticizer [21,22,23,24]. For the low modulus PMMA developed for vertebroplasty, 12 vol% LA has mostly been used [23,24]. The LA-PMMA could have a potential also in discoplasty, for overcoming the mismatch in endplate–PMMA modulus and for reducing the risk for endplate fracture and subsidence. To reach a higher mobility in the spine than complete fusion, it might also be interesting to evaluate PMMA cements with an even higher amount of LA. However, it is unknown how materials with different stiffness affect the spinal mobility after discoplasty.

In development of biomaterials and implants for spinal treatment, biomechanical experiments can be challenging and time consuming as well as give a large variation in results due to natural variability. Computational finite element (FE) models of vertebral segments have previously been found useful for comparing techniques for treatment with spinal fusion, spinal cages, laminectomies, etc. [25,26,27,28,29], but no studies have been found where FE-models simulate the biomechanics of discoplasty. FE-models could provide a great opportunity to get a first understanding of the biomechanical potential of standard PMMA and low-modulus PMMA in discoplasty.

There were two aims of this study. First, to compare standard PMMA and PMMA with lower E-modulus in a biomechanical lumbar spine discoplasty model. A secondary aim was to experimentally obtain PMMA cements with lower modulus than previous PMMA-based cements. We hypothesize that both standard PMMA and low modulus PMMA will stabilize the spinal segment, and that the low modulus PMMA will result in decreased stresses on the endplates.

## 2. Materials and Methods

### 2.1. Compressive Properties of Low-Modulus PMMA

#### 2.1.1. Material Preparation and Storage

A commercial PMMA bone cement (V-Steady, G21 S.r.l., San Possidonio, Italy) was used as a control and base cement. This bone cement consists of two components, the powder contains pre-polymerized PMMA, benzoyl peroxide, and zirconium dioxide (ZrO_2_), and the liquid contains mainly methyl methacrylate and small amounts of N,N-dimethyl-p-toluidine and hydroquinone. V-Steady was also used as base cement in the modified low-modulus bone cements, which in addition contained linoleic acid (LA) as an additive. The linoleic acid (Evonik Industries, Essen, Germany) had been heat sterilized before use [23].

The V-Steady was prepared by mixing the powder and the liquid in a glass mortar with a spatula for 45 s at room temperature (according to the instructions from the manufacturer). The liquid component of V-Steady was modified in order to make three types of low-modulus bone cements, with 12 vol%, 16 vol% and 20 vol% LA. The amounts were based on preliminary studies where they gave a satisfactory decrease in modulus, down to vertebral trabecular bone levels, and a further increase would give an excessive decrease in yield strength of the material. Here, the V-Steady liquid phase was mixed with the LA, and the liquid was then mixed with the powder in a glass mortar with a spatula for 45 s at room temperature. The groups of the modified cement are hereinafter referred to as LA12, LA16 and LA20.

After mixing, the resulting paste was poured into cylindrical molds (ø = 6 mm and h = 12 mm) and left to set in air at 37° (Memmert UN75Plus, Memmert GmbH, Schwabach, Germany). After one hour, the molds were placed in phosphate buffered saline (PBS tablets, P4417, Sigma-Aldrich Merck, Darmstadt, Germany) for 24 h. Thereafter, the specimens were taken out from the molds and tested (the 24 h storage group) or stored for longer periods of time (the 48 h, 1 week, 2 weeks, 4 weeks and 8 weeks storage groups). After storage, the end surfaces of the specimens were polished plane using wet silicon carbide paper.

#### 2.1.2. Quasi-Static Compression Testing

The compression tests were performed in a universal testing machine (AGS-X, Shimadzu Corp., Kyoto, Japan). The specimens were loaded parallel to their longitudinal axis at a crosshead speed of 20 mm/min, in accordance with ISO 5833 [30]. A load cell with 5 kN capacity was used, and the displacement was measured with an optical encoder. The displacement data were corrected for machine compliance. Subsequently, stress–strain curves were determined for all specimens. The compressive strength (CS) was calculated from the 2% offset load or the upper yield-point, whichever occurred first, in accordance with ISO 5833 [30]. The elastic modulus (E) was calculated from the slope of the linear part of the stress–strain curve.

#### 2.1.3. Statistical Analysis

Statistical analyses were performed in R (version 3.5.2) [31]. First, Shapiro–Wilk and Levene’s tests were conducted to assess the normality and the homoscedasticity of the data. Since Shapiro–Wilk and Levene’s tests were significant for a probability value *p* < 0.05, a robust two-way ANOVA was performed to determine differences in material properties among the groups with different amount of LA and storage times [32]. A Tukey’s post hoc test was then performed, and statistically significant differences were confirmed for a probability value *p* < 0.05.

### 2.2. Discoplasty in a Lumbar Spine Computational Model

#### 2.2.1. The Open-Access Human Lumbar Spine Model

The open-access human lumbar spine model has been thoroughly described elsewhere [28,33,34,35]. The model was implemented in the open-source software FEBio Studio version 1.0 (www.febio.org, accessed on 18 December 2021) by Finley et al. [33]. Several spinal models are available in FEBio. For this project, the lower part of the lumbar spine (L4-L5) was chosen, since this seems to be one of the more common levels treated by discoplasty in the clinics [7]. The geometry of the model was constructed from CT scans of a 49-year-old cadaveric specimen. A brief overview of the model is provided below for clarity and context.

**Figure 1:**
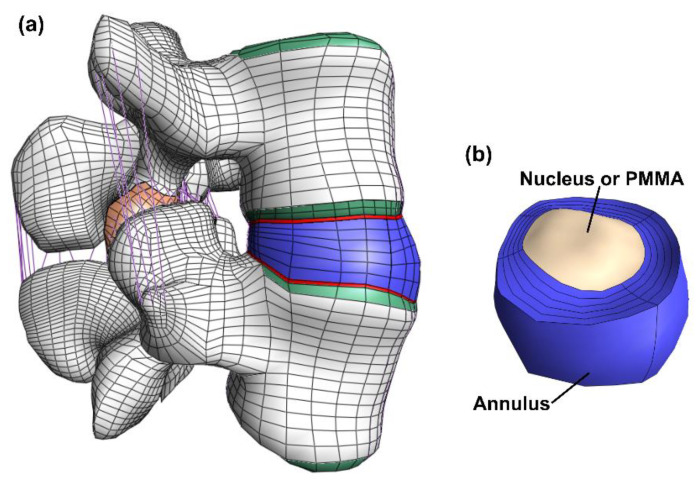
Overview of the model. The full model (**a**) including bone (white), bony endplate (green), cartilaginous endplates (red), facet joint cartilage (orange), ligaments (purple) and the disc (blue). The disc (**b**) where both the annulus (blue) and the nucleus (beige) is visible. In the discoplasty model the nucleus is exchanged with PMMA cement.

The model consisted of cortical bone, trabecular bone, posterior bone, vertebral bony endplates, cartilaginous endplates, facet joint cartilage and intervertebral disc (nucleus pulposus and annulus fibrosus). (Figure 1). The geometry was meshed by linear hexahedral elements (hex8 in FEBio). Table 1 shows the constitutive models and material properties used for each part [33,34,36,37,38]. A neo-Hookean constitutive model (linearly elastic for small strains) was used for the posterior bone, trabecular bone, vertebral bony and cartilaginous endplates, facet cartilage, and nucleus pulposus. The cortical bone was modeled by an orthotropic elastic model. For the annulus fibrosus, a Holmes-Mow constitutive model was used for modeling the matrix and an exponential-power law modeled the fibrous part of the annulus. The seven major ligaments of the lumbar spine were modeled by non-linear tension-only spring elements and assigned force-displacement curves from literature [33,34,37].

The most inferior and the most superior vertebral bony endplates were approximated as rigid bodies. For each model, all displacements at the most inferior vertebral endplate were fixed. At the most superior vertebral endplate, a moment of 7.5 Nm was applied in flexion, extension, lateral bending, and axial rotation. The loading has been recommended in testing of lumbar spines with pure moments [39]. In addition, an expansive pressure load of 0.1 MPa was applied to the nucleus pulposus, representing the lower range of physiological pressure [40]. A sliding contact (no friction) was defined at the facet joints.

As a first step in the current study, the models were reproduced in a more recent version of the FEBIO software (FEBio Studio 1.5.0) [41]. The range of motion, defined as the Euler angles at the most superior vertebral bony endplate, was compared to previous results. Moreover, the distribution of the first principal strain in the cortical bone was compared to previous results.

#### 2.2.2. Modification for Discoplasty

The present study focused on discoplasty, its resulting effect on the ROM and stresses at the endplates. Therefore, a number of modifications were made to the lumbar spine model. First, the nucleus was replaced by a material representing the different PMMA bone cements (V-Steady, LA12, LA16 and LA20). The nucleus in the model had a volume of 4.6 mL, similar to reported volumes of PMMA after discoplasty [4,6]. The bone cements were modeled as a linear elastic isotropic material. The material properties for all cements were obtained in compression tests after 24 h storage in this study.

Mesh convergence have been evaluated in one of the earlier studies on the same model [34]. However, they evaluated the strain energy density and ROM. In the present study, the stresses in the endplate are also of interest. A separate mesh convergence study was carried out for this purpose. The average endplate von Mises stress was evaluated, hereinafter referred to as AEVM. In the models where the mesh had been modified, a tied contact was used to connect the new endplate to the cartilage endplate and the vertebra. ROM and AVME was evaluated for the original model (FEorg), a model with the original endplate mesh but implemented contact (FEcon), a model with one mesh refinement step (FEfine1), a model with the same mesh refinement but 20-node hexahedral elements (FEfine1hex), and a model with two mesh refinement steps (FEfine2). The analysis of stresses was focused on the inferior L4 vertebral bony endplate. The reason was that FEorg, with PMMA (V-steady) discoplasty, demonstrated higher AEVM in the inferior L4 endplate as compared to the superior L5 endplate for all load cases. Mesh convergence was checked for the models with a healthy nucleus and for the discoplasty model with standard PMMA (V-steady). The number of elements in the final converged mesh was 36,444. All models were solved using FEBio Studio version 1.5.0 [41].

## 3. Results

### 3.1. Compressive Material Properties for Low-Modulus PMMA

**Figure 2:**
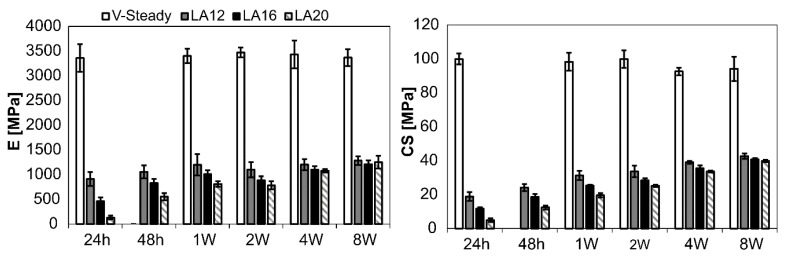
Results for E-modulus (**left**) and compressive yield strength (**right**). The results over time (24 h, 48 h, 1 week, 2 weeks, 4 weeks and 8 weeks) presented for V-Steady, LA12, LA16 and LA20.

The mechanical properties for all cement types over time can be seen in Figure 2. The detailed results are given in Table 2 (E-modulus) and Table 3 (CS). Both E-modulus and compressive strength differed significantly for V-steady as compared to the modified cements (LA12, LA16 and LA20), at all storage timepoints. The E-modulus for V-Steady ranged from 3360 ± 277 MPa at 24 h to 3473 ± 101 MPa at 4 weeks, with no significant change between the storage timepoints. There was no significant change in compressive strength for V-steady between time points, the CS ranged from 93 ± 7 MPa at 4 weeks, to 100 ± 3 at 24 h.

At 24 h storage, the E-modulus was decreased by 73%, 86% and 96% for LA12, LA16 and LA20, as compared to V-steady, i.e., the more LA being used the lower the E-modulus at 24 h storage. At 8 weeks the same numbers were 62%, 64% and 63% for LA12, LA16 and LA20, as compared to V-steady, i.e., at 8 weeks, the modified cements had similar E-modulus, independently of amount of LA added initially. The increase in E-modulus from 24 h storage to 8 weeks storage was: 131 ± 39 MPa to 1253 ± 130 MPa for LA20, and 908 ± 140 MPa to 1284 ± 87 MPa for LA12.

**Figure 3:**
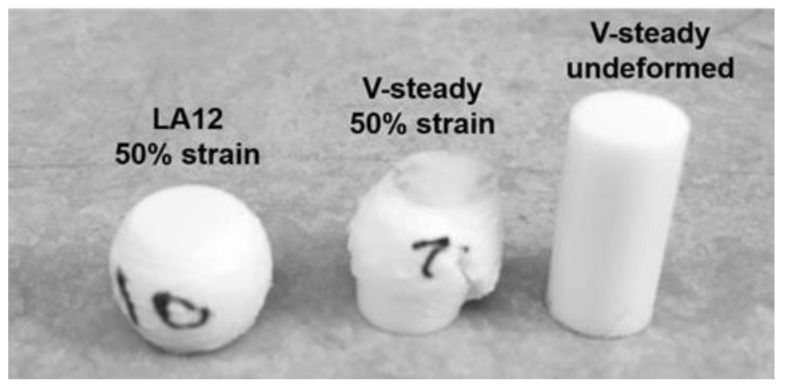
A deformed LA12 and a fractured V-steady specimen after compression test to 50% strain after 4 weeks of storage. A V-steady sample before compression test is included as a reference.

The mode of deformation was very different for the V-steady specimens as compared to the modified cements. All modified cements could be deformed up to 50% strain without any visible fractures throughout the specimens. In contrast, the V-Steady specimens fractured before 30% strain. Examples of the differences in deformation modes after 4 weeks can be seen in Figure 3.

### 3.2. Discoplasty in a Lumbar Spine Computational Model

The ROM results reported by Finley et al. could be reproduced. Moreover the contour plot for the first principal cortical bone strain was reproduced [33].

Mesh convergence was checked by comparing the finest mesh (FE_fine2_) with the other models. In addition, FE_org_ was compared to the model with contact (FE_con_) to look at the influence of implementing a tied contact. The mesh did not affect the ROM, all models (FE_org_, FE_con_, FE_fine1,_ FE_fine1hex_ and FE_fine2_) had less than 2% difference in all load cases (flexion-extension, lateral bending and axial rotation). For the AEVM, FE_org_ and FE_con_ had less than 2% difference. However, the mesh at the vertebral bony endplate had an effect. FE_org_ compared to FE_fine2_ had up to 11% difference in AEVM. However, after one step of mesh refinement (FE_fine1_) the difference in AEVM compared to FE_fine2_ was below 3%. In addition, no visible differences were found in the contour plots for the von Mises stress between FE_fine1_ and FE_fine2_. Further details about the mesh convergence are presented in the supplementary material, Tables S1 and S2. Due to these results, FE_fine1_ was used in the simulations presented below.

**Figure 4:**
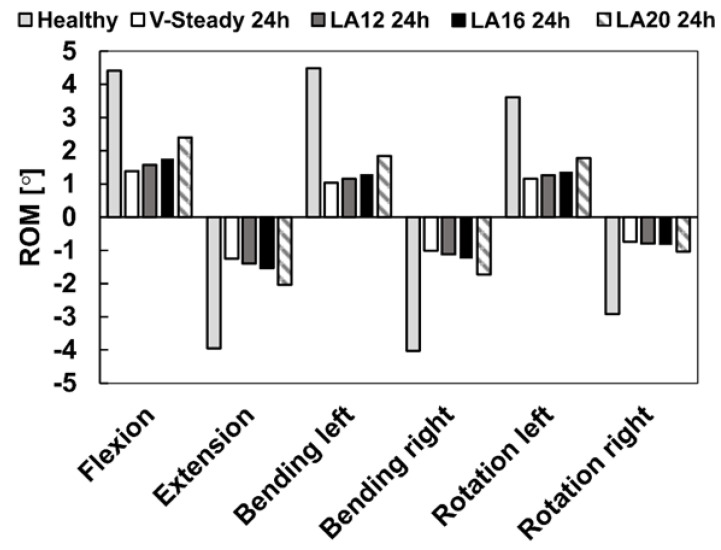
Range of motion in flexion, extension, lateral bending (left and right) and rotation (left and right). The results are shown for the healthy model and the discoplasty models with the different bone cements: V-Steady, LA12, LA16 and LA20.

**Figure 5:**
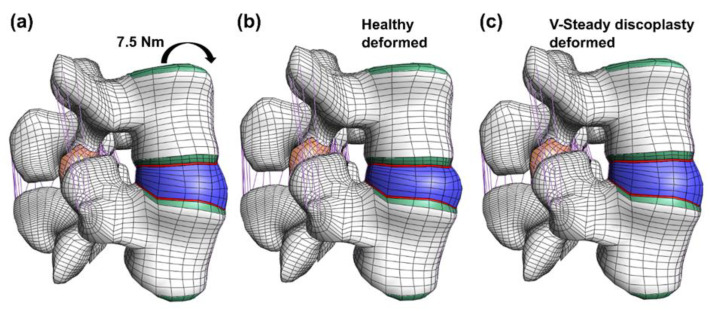
Undeformed and deformed models in flexion. The undeformed model (**a**) is compared to two models loaded by a 7.5 Nm moment in flexion: the healthy model (**b**) and the V-Steady discoplasty model (**c**).

The healthy model was compared to the discoplasty models with the different PMMA cement types (V-steady, LA12, LA16 and LA20). Figure 4 presents the ROM for all models in all loading scenarios. For the healthy model, flexion and left bending had the largest ROM (4.4° and 4.5°, respectively). The smallest ROM for the healthy model was seen in right rotation (2.9°). All discoplasty models showed the same trend, a large reduction in range of motion. The discoplasty models had the largest ROM in flexion and extension. The smallest ROM for the discoplasty models was seen in right rotation, just as for the healthy models. For all the discoplasty models, the reduction as compared to the healthy model was determined in percent. The V-Steady discoplasty model had a reduction from 68% in left rotation up to 77% in left bending. For the modified cements the reductions were from 65% in left rotation up to 74% in left bending for LA12, from 60% in flexion up to 71% in left bending for LA16, and from 46% in flexion up to 65% in right rotation for LA20. Figure 5 shows a comparison between the healthy and the V-Steady discoplasty, undeformed and deformed, models in flexion (69% reduction in ROM).

**Figure 6:**
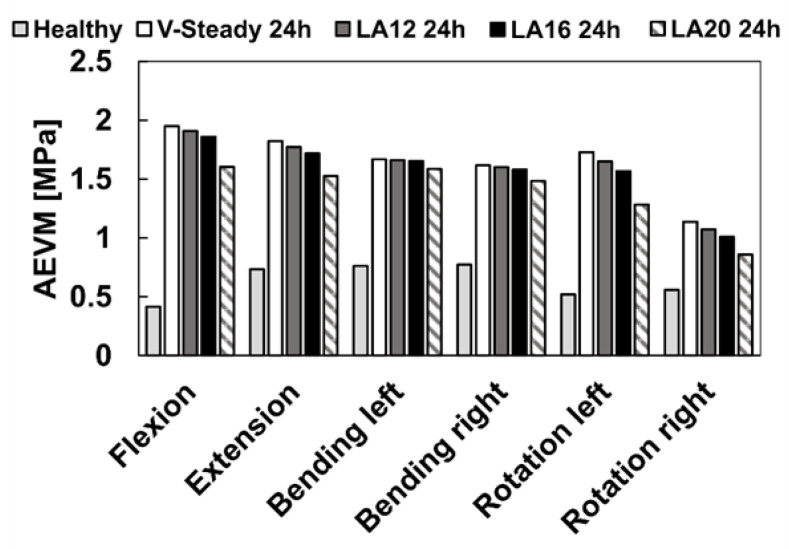
Average endplate von Mises stress in flexion, extension, lateral bending (left and right) and rotation (left and right). The results are shown for the healthy model and the discoplasty models with the different bone cements: V-Steady, LA12, LA16 and LA20.

**Figure 7:**
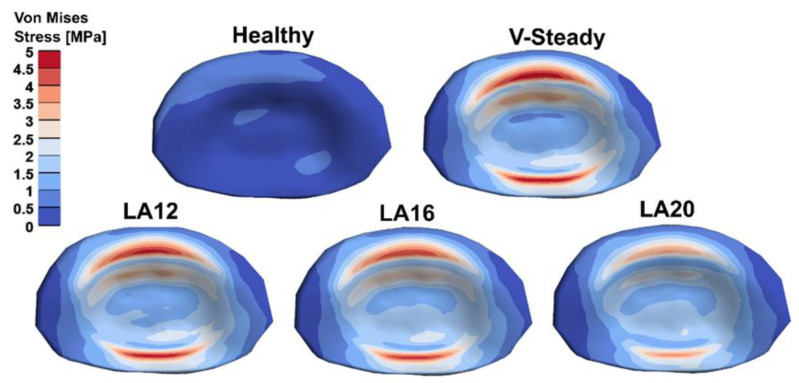
Von Mises stress distribution in the L4 inferior endplate in flexion for the healthy model and the discoplasty models with the different bone cements: V-Steady, LA12, LA16 and LA20.

**Figure 8:**
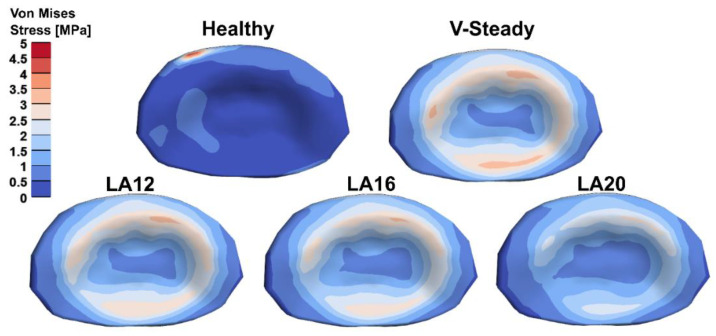
Von Mises stress distribution in the L4 inferior endplate in left rotation for the healthy model and the discoplasty models with the different bone cements: V-Steady, LA12, LA16 and LA20.

The AEVM was evaluated at the L4 inferior endplate for all models in all loading scenarios (Figure 6). All discoplasty models had a higher AEVM compared to the healthy model. Comparing the healthy model to the V-steady discoplasty model, the increase was from 369% in flexion, to 103% increase in rotation. In the models with the modified cements (LA12, LA16 and LA20), the AEVM decreased as compared to the V-steady discoplasty model. Compared to V-steady discoplasty, LA12 had up to 6% lower AEVM (in right rotation), LA16 had up to 11% lower AEVM (in right rotation), and LA20 had up to 26% lower AEVM (in left rotation). Figure 7 shows the von Mises stress distribution in the L4 inferior endplate in flexion for the healthy model and the discoplasty models with the different bone cements (V-Steady, LA12, LA16 and LA20). Figure 8 shows the same but in left rotation. In both Figure 7 and Figure 8, an increase in overall stress is seen for all discoplasty models. As compared to V-steady the modified cements had smaller areas of the highest stress. The LA20 model had the lowest stresses out of the modified cement models, as expected. In rotation (Figure 8), it can be noticed that a higher stress was seen also for the healthy model. Nevertheless, the higher stress was located on the outer edge of the endplate and on a relatively small area.

## 4. Discussion

This study developed low modulus PMMA cements with higher amounts of linoleic acid (16 vol% and 20 vol%) than what has been previously used (12 vol%). As expected, the E-modulus at 24 h storage was lower for the PMMA cements with a higher amount of linoleic acid (462 ± 78 MPa for LA16 and 131 ± 39 MPa for LA20), as compared to the previously tested (908 ± 140 MPa for LA12). Nevertheless, after storage at 37° in PBS for longer periods, both the E-modulus and CS increased for the modified cements. The use of the developed cements was investigated by computational lumbar spine models with a healthy disc, or a disc treated by discoplasty. All discoplasty models showed the same trend—a large reduction in ROM as compared to the healthy model. The largest reduction in ROM was seen for the discoplasty model with V-steady (77% reduction), and the smallest reduction of ROM in the LA20 model (45% reduction). Compared to V-steady discoplasty, LA20 discoplasty had up to 26% lower average von Mises stress in the endplate.

Previous studies have reported cement CS similar to the current study. In the present study, a CS of 93 ± 7 MPa and 39 ± 1 MPa was obtained after 4 weeks, for V-steady and LA12, respectively. The same materials (LA12 and V-steady) and test conditions, previously gave a CS of 92 ± 17 MPa and 37 ± 1 MPa [23,24]. In contrast, there was a substantial difference between the E-modulus obtained in the current study as compared to previous studies. In the present study, E-modulus of 3434 ± 282 MPa and 1202 ± 113 MPa were obtained after 4 weeks for V-steady and LA12, respectively. The same materials (LA12 and V-steady) and test conditions previously gave an E-modulus of 2070 ± 103 MPa and 948 ± 64 MPa [23,24]. The results of the current study are more in line with the material properties data given by the manufacturer of V-Steady [42]. The higher E-modulus in the current study should be attributed to previous studies not conducting a correction for machine compliance of the material tester. It can be noted that for V-steady the material properties did not change significantly over time. For LA12 there were significant changes in both E-modulus and CS (24 h to 1 week). However, between 4 weeks and 8 weeks there were fewer changes, and they seemed to plateau. For the higher amounts of linoleic acid, LA16 and LA20, the properties changed significantly over time up to 8 weeks. At 8 weeks the E-modulus (1212 ± 78 MPa for LA16 and 1253 ± 130 MPa for LA20) were almost at the same level as for LA12 (1284 ± 87 MPa), likely due to a combination of LA diffusing out of the cement and a decrease in free volume over time [23]. A higher initial amount of LA did not seem to have a significant effect on the reduction of the polymerization rate. However, it is not clear if the change in material properties over time is a large problem since the bone will also heal and remodel during the healing phase. Nevertheless, more testing needs to be conducted before these cements could be considered for in vivo studies. Monomer release and handling properties such as setting time, polymerization and glass transition temperature would all need verification. While a release of small amounts of LA would not be a concern, a previous study has showed a higher monomer release for LA12 than for V-Steady [24]. Previous studies have also reported on the handling properties for LA12 [23]. As compared to standard PMMA, the 12% LA PMMA showed no significant change in setting time, but a longer working (injectability) time. The PMMA with higher amounts of LA could possibly slow down the polymerization further and thereby increase these effects. However, all handling properties should be investigated in future studies.

The use of the developed cements was investigated by computational lumbar spine models with a healthy disc, or a disc treated by discoplasty. All discoplasty models showed the same trend—a large reduction in ROM as compared to the healthy model. In contrast, the only previously published experimental discoplasty study did not see a significant change in range of motion or stiffness following discoplasty [15]. However, a decrease in strain at the disc was observed [15]. Nevertheless, more biomechanical studies of the biomechanics of discoplasty are needed. The models provided a way to compare the potential effect of using PMMA with different stiffnesses. Compared to V-steady discoplasty, LA20 discoplasty had up to 26% lower average von Mises stress in the endplate. A previous study used a similar modeling approach and loading conditions to model L3-L5 segments before and after spinal fusion [29]. They obtained ROMs below 0.5° for the models with fusion, which is lower than what we obtained after discoplasty: around 1º for LA12 and around 2° for LA20. It should be noted that their healthy models had ROMs around 2–3°, whereas our models had ROMs between 3–4° [29]. However, several differences exist between the models in terms of, e.g., geometry, contact conditions and material data. In future studies, it would be of high interest to compare discoplasty and fusion in the same model to further investigate differences between the treatments.

The main purpose with the discoplasty models was to get a first understanding for the biomechanical effects of discoplasty, and the effect of using bone cements with different stiffnesses. To the authors knowledge, no previous computational studies exist on discoplasty. Discoplasty is a relatively new clinical procedure that was developed in the clinics [4,5,6,9]. In addition, only one experimental biomechanics study exists as previously mentioned [15]. Therefore, both clinical and experimental information about discoplasty is limited. Several assumptions and simplifications were therefore made in the models. However, the models could be further developed in future studies. One of the largest limitations is the simplified interface between the PMMA and surrounding tissues. In this study, the PMMA and surrounding tissues were modeled as tied. It is well known that in reality the interface is more complex [43,44,45]. A recent study has made interesting observations on clinical CT images from discoplasty patients. That study made patient-specific volumetric measurements of the amount of injected PMMA and correlated this to the volumetric increase of the spinal canal after discoplasty [46]. In future studies, clinical information, e.g., volume of injected PMMA or PMMA–tissue interface, could be added to improve the FE-models. In addition, it would be of interest to explore patient-specific discoplasty FE-models. Here, it would be highly relevant to obtain the grayscale values from the CT images and relate those to the bone mineral density. This would give a more accurate representation of the local material properties, e.g., in the endplates. In the case of using patient-specific data, data more representative of patients that are normally treated by discoplasty would be optimal, and allow for studying optimal materials for certain conditions, such as osteoporotic patients or patients with sclerotic endplates (giving rise to a higher or lower risk for subsidence, respectively). However, this would also require experimental cadaver or animal ex-vivo studies for validation to achieve a high enough resolution of the images.

Moreover, the present study only used an L4-L5 model. Future studies should aim to investigate the full lumbar spine in order to also look at the adjacent segments. Lastly, since the stress at the endplates is of high interest, the endplates should be more carefully modeled. In reality the endplates have spatially varying material properties, with weaker bone in the center and stronger bone at the peripheral parts [25,47]. This could also be addressed through relating CT data to bone mineral density in a patient-specific model. Despite the above-mentioned limitations, the models provided a relevant comparison between the cement types, and between a healthy disc and a disc treated by discoplasty. In future studies, computational models could be used as a tool to find a cement for discoplasty in degenerated spinal segments with optimal mechanical properties for satisfactory reduction of painful movement and intraforaminal neural compression.

## 5. Conclusions

In conclusion, a PMMA cement with lower E-modulus could be obtained by increasing the amount of LA. Up to 20 vol% LA was used and an E-modulus of 131 ± 39 MPa was achieved after 24 h. However, the E-modulus increased with time. If the properties of the PMMA should remain, ways of stabilizing the effect of LA in the PMMA should be investigated.

The finite element models showed that low modulus PMMA could be promising for use in discoplasty. The average stress at the endplate was higher for all discoplasty models than for the healthy model, but the stresses were reduced for cements with higher amounts of LA. The study indicates that low-modulus PMMA is promising for discoplasty from a mechanical viewpoint. However, validation experiments of important details such as the cement–endplate attachment are needed before giving the clinical setting further consideration.

## Figures and Tables

**Table 1:** Constitutive models and material properties for the different parts in the model.

Part	Constitutive Model	E-Modulus [MPa]	Poisson’s Ratio
Cortical bone	Orthotropic elastic	E_1_ = 8000	ν_12_ = 0.4
		E_2_ = 8000	ν_23_ = 0.3
		E_3_ = 12,000	ν_31_ = 0.35
Trabecular bone	Neo-Hookean	E = 100	ν = 0.2
Posterior bone	Neo-Hookean	E = 3500	ν = 0.3
Vertebral endplate	Neo-Hookean	E = 1000	ν = 0.3
Cartilaginous endplate	Neo-Hookean	E = 23.8	ν = 0.42
Nucleus pulposus	Neo-Hookean	E = 1	ν = 0.49
Annulus matrix		E = 1	ν = 0.4
		β = 3.4	
Annulus fibers		α = 65	
		β = 2	
		ξ = 0.296	
Facet cartilage	Neo-Hookean	E = 30	ν = 0.4

**Table 2:** E-modulus over time for the different PMMA-cements.

E-Modulus [MPa]						
	24 h	48 h	1 Week	2 Weeks	4 Weeks	8 Weeks
V-steady	3360 ± 277	-	3401 ± 143	3473 ± 101	3434 ± 282	3368 ±173
LA12	908 ± 140	1056 ± 131	1198 ± 213	1097 ± 153	1202 ± 113	1284 ± 87
LA16	462 ± 78	829 ± 82	1009 ± 79	883.8 ± 80	1099 ± 68	1212 ± 78
LA20	131 ± 39	553 ± 71	812 ± 56	780 ± 83	1077 ± 39	1253 ± 130

**Table 3:** Compression strength over time for the different PMMA-cements.

CS [MPa]						
	24 h	48 h	1 Week	2 Weeks	4 Weeks	8 Weeks
V-steady	100 ± 3	-	98 ± 5	100 ± 2	93 ± 7	94 ± 2
LA12	19 ± 3	24 ± 2	31 ± 3	34 ± 3	39 ± 1	43 ± 2
LA16	12 ± 1	19 ± 2	25 ± 1	28 ± 1	36 ± 2	41 ± 1
LA20	5 ± 1	12 ± 1	20 ± 1	25 ± 1	34 ± 1	40 ± 1

## Data Availability

The data presented in this study are available on request from the corresponding author.

## Supplementary Materials

The following supporting information can be downloaded at: https://www.mdpi.com/article/10.3390/jfb13010018/s1, Table S1: Comparison of absolute percent difference in ROM in different directions for the different meshes. The three first lines present the healthy models, and the three below the PMMA discoplasty models.; Table S2: Comparison of absolute percent difference in AEVM in different directions for the different meshes. The three first lines present the healthy models, and the three below the PMMA discoplasty models.
