# Supplementary material for: Low-Modulus PMMA Has the Potential to Reduce Stresses on Endplates after Cement Discoplasty
Source: J Funct Biomater. 2022 Feb 4;13(1):18. doi: 10.3390/jfb13010018 (PMC8883899; doi:10.3390/jfb13010018)
Supplement: Supplementary file 1 [file jfb-13-00018-s001.zip › jfb-1539479-supplementary.pdf]

S1: Supplementary information

Mesh convergence

Data regarding mesh convergence for the lumbar spine discoplasty models are available below in Table S1 and Table S2.

Table S1. Comparison of absolute percent difference in ROM in different directions for the different meshes. The three first lines present the healthy models, and the three below the PMMA discoplasty models.

	Flexion	Extension	Bending left	Bending right	Rotation left	Rotation right
**FE_{org} vs. FE_{con}**	0.02%	1.89%	0.21%	0.90%	0.10%	0.09%
**FE_{con} vs. FE_{fine2}**	0.001%	1.35%	0.09%	0.64%	0.03%	0.004%
**FE_{fine1} vs. FE_{fine2}**	0.004%	0.13%	0.04%	0.07%	0.02%	0.02%
**PMMA: FE_{org} vs. FE_{con}**	0.09%	1.55%	0.16%	0.19%	0.06%	0.11%
**PMMA: FE_{con} vs. FE_{fine2}**	1.66%	1.42%	2.04%	1.89%	0.42%	0.33%
**PMMA: FE_{fine1} vs. FE_{fine2}**	0.47%	0.43%	0.62%	0.56%	0.10%	0.07%

Table S1. Comparison of absolute percent difference in AEVM in different directions for the different meshes. The three first lines present the healthy models, and the three below the PMMA discoplasty models.

	Flexion	Extension	Bending left	Bending right	Rotation left	Rotation right
**FE_{org} vs. FE_{con}**	0.46%	1.52%	0.44%	0.93%	0.30%	0.22%
**FE_{con} vs. FE_{fine2}**	3.73%	11.22%	5.89%	6.10%	7.97%	6.99%
**FE_{fine1} vs. FE_{fine2}**	1.35%	4.02%	1.93%	2.49%	2.48%	2.16%
**PMMA: FE_{org} vs. FE_{con}**	0.39%	1.55%	0.41%	0.97%	0.27%	0.18%
**PMMA: FE_{con} vs. FE_{fine2}**	10.43%	9.60%	12.44%	11.87%	2.87%	2.93%
**PMMA: FE_{fine1} vs. FE_{fine2}**	2.91%	2.52%	3.23%	3.06%	0.91%	0.91%
